# Supplementary material for: Development of the macaque face-patch system
Source: Nat Commun. 2017 Mar 31;8:14897. doi: 10.1038/ncomms14897 (PMC5381009; doi:10.1038/ncomms14897)
Supplement: Supplementary Information — Supplementary figures, supplementary table, and supplementary references. [file ncomms14897-s1.pdf]

Supplementary Table 1.

B1 136d N=19
B1 180d N=28
B1 277d N=30
B1 375d N=23
B1 382d N=27
B1 411d N=51
B1 564d N=33

B2 48d N=6
B2 132d N=9
B2 153d N=20
B2 160d N=30
B2 174d N=14
B2 209d N=14
B2 265d N=21
B2 391d N=12

B3 24d N=6
B3 38d N=7
B3 81d N=24
B3 88d N=24
B3 102d N=26
B3 123d N=21
B3 178d N=17
B3 207d N=7
B3 221d N=10
B3 252d N=16
B3 270d N=24
B3 284d N=20
B3 312d N=27
B3 403d N=12
B3 410d N=10
B3 438d N=16
B3 445d N=14
B3 508d N=7
B3 515d N=14
B3 529d N=18
B3 557d N=14
B3 655d N=14
B3 676d N=24
B3 774d N=17

B4 16d N=5
B4 30d N=5
B4 73d N=17
B4 80d N=13
B4 115d N=20
B4 170d N=23
B4 199d N=20
B4 213d N=11
B4 244d N=18
B4 262d N=25
B4 276d N=23
B4 304d N=16
B4 395d N=14
B4 402d N=11
B4 430d N=11
B4 437d N=10
B4 500d N=12
B4 507d N=16
B4 521d N=17
B4 549d N=13
B4 647d N=14
B4 668d N=22
B4 766d N=10
B4 787d N=15
B4 836d N=14

Supplementary Table 1. Number of blocks of each category accepted for analysis for each scan session for each monkey (same number of blocks accepted for each category).

Supplementary Figures

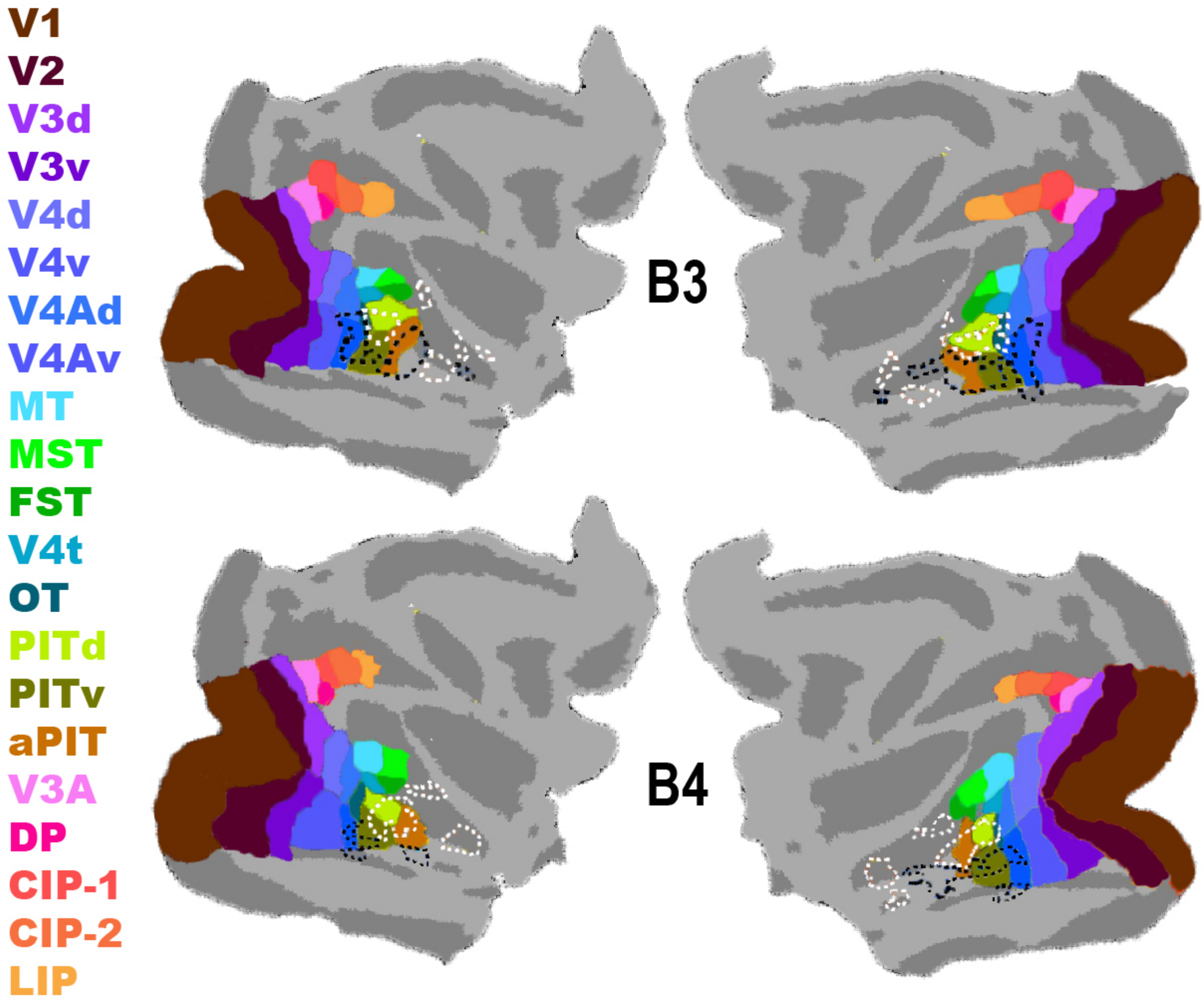

Supplementary Figure 1. Face and Object ROIs overlaid on visual areas. Visual areas were determined using phase-encoded retinotopic mapping; see Arcaro et al.[1] for methods; in monkeys B3 and B4 at >2 years of age. Visual areas are in general agreement with prior studies [2,3] with the addition of a retinotopic map of contralateral space in the anterior-most portion of PIT (aPIT). White dotted lines indicate face-selective ROIs and black dotted lines indicate object ROIs, both calculated for the same monkeys at ages 284 and 276 days old respectively; see top center of Figure 2.

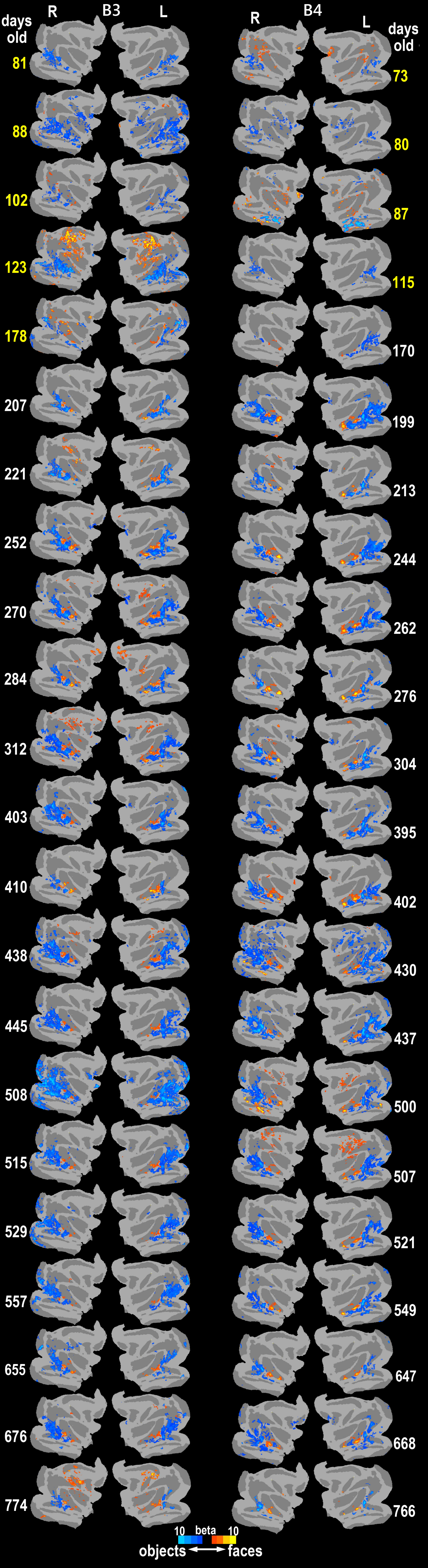

days old
R
B3
L
R
B4
L
days old
81
88
102
123
178
207
221
252
270
284
312
403
410
438
445
508
515
529
557
655
676
774
73
80
87
115
170
199
213
244
262
276
304
395
402
430
437
500
507
521
549
647
668
766
10
beta
10
objects
faces

Supplementary Figure 2. Development of face selectivity. Non-overlapping maps (same data as in Fig. 2) of activations (beta coefficients) for the contrast faces-minus-objects for each static-image scanning session for monkeys B3&B4 projected onto the standard F99 macaque brain flat map [4,5]. Each monkey's age in days is indicated beside each map. Where the age is indicated in white, the beta coefficients were thresholded at p<=0.05, FDR-corrected; where the age is indicated in yellow, there were no significant faces>objects activations at p<0.05 in the STS, so the beta coefficients were thresholded at p<=0.25, FDR-corrected.

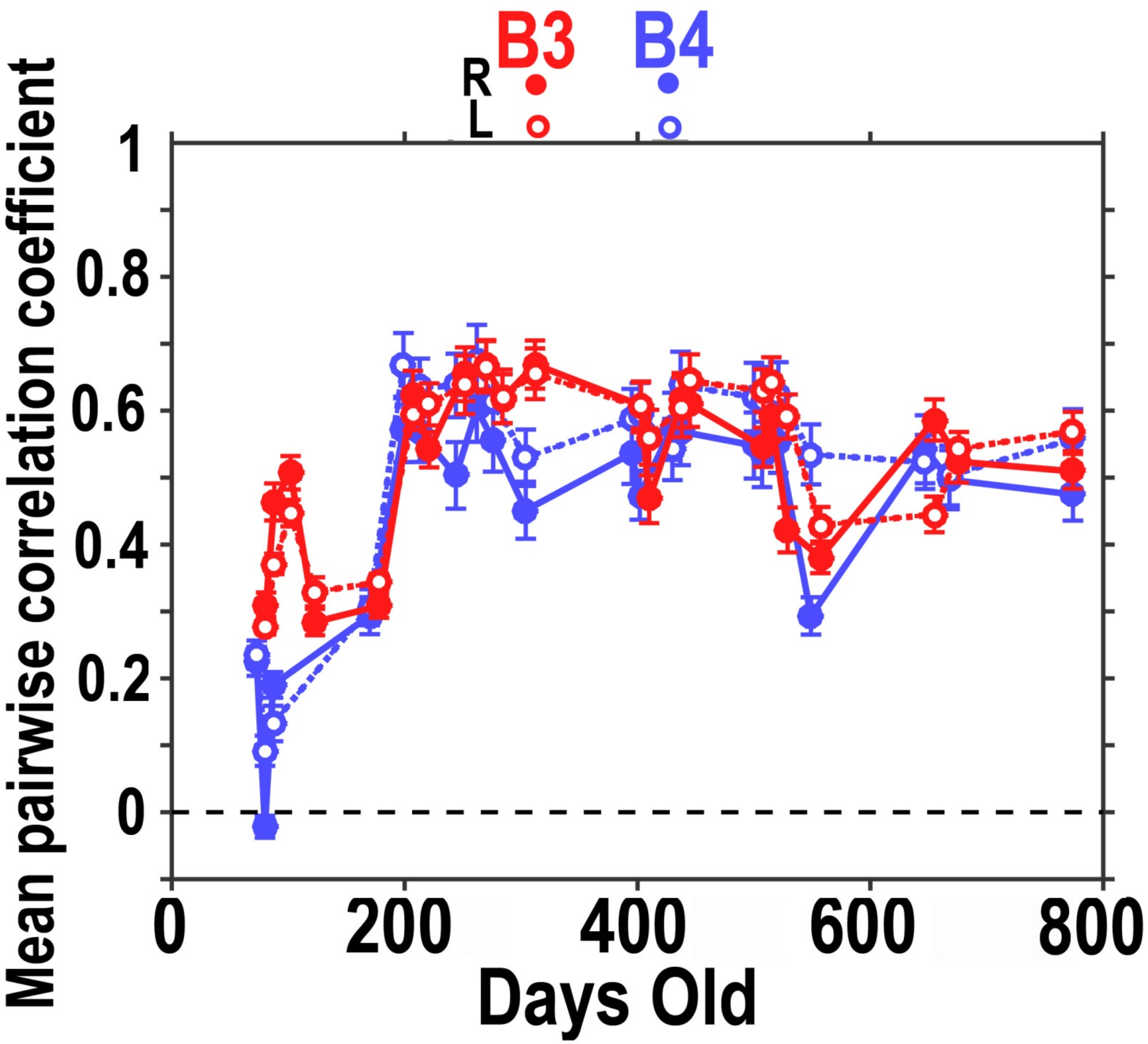

Supplementary Figure 3. Spatial correlations between faces-minus-object activations for monkeys B3 and B4. Symbols show mean pairwise correlation coefficients between each date and all other dates of the spatial pattern of faces-minus-objects (unthresholded) beta coefficients in the lower bank and lip of the STS , right and left hemispheres, as indicated. Each symbol represents the mean correlation (± sem) of the spatial faces-minus-objects activation pattern with all other dates for that hemisphere for that monkey. The STS ROI was the same ROI as was used for Figure 7 and Supplementary Figure 8. This anatomical ROI encompassed the entire anterior-to-posterior length of the lower lip of the STS from the anterior tip to the anterior border of V4.

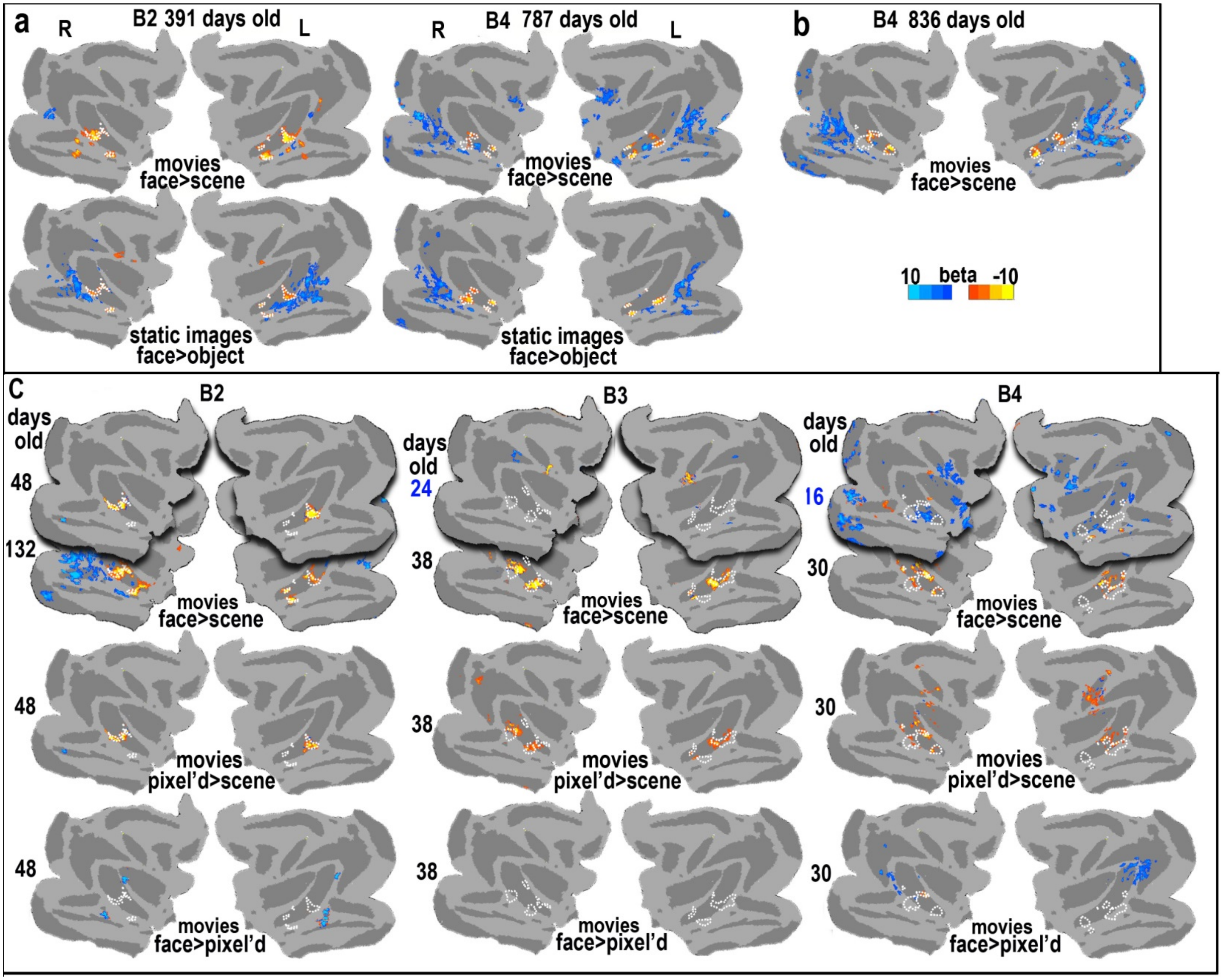

Supplementary Figure 4. Responses to movies in juveniles and infants. a) Justification for using movies to reveal the face-patch system: Two monkeys (older than the age when they exhibited significant static-image face patches) viewed either movies (face-movies/scene-movies) or static-image blocks (faces/objects) in the same scan session. Maps show activations thresholded at p<0.05 FDR corrected for the contrast faces-minus-scenes movies (above) and faces-minus-objects static images (below). Dotted lines outline the significant faces>objects regions in the STS from the same scan session. b) Monkey B4 at 836 days old viewed movies, and we compared the activations to movies to static-image activations in the same monkey on a different date. White outlines indicate the locations of significant faces>objects (static images) in the STS from the same monkey at 276 days old. c) Activations for the contrast faces-minus-scenes movies (above) for three infant monkeys at two ages each. (below) Maps for the contrast faces-minus-pixelated faces from the youngest sessions in each monkey that showed significant faces>scenes movie activations. Where the ages are indicate in black activations were thresholded at p<0.05 FDR corrected; where the ages are in blue, activations were thresholded at p<0.25 FDR corrected. Dotted white outlines indicate the locations of significant faces>objects (static images) activations from the same monkey for B2 at 391 days old; for B3 at 284 days old; for B4 at 276 days old (same data as in Fig. 3).

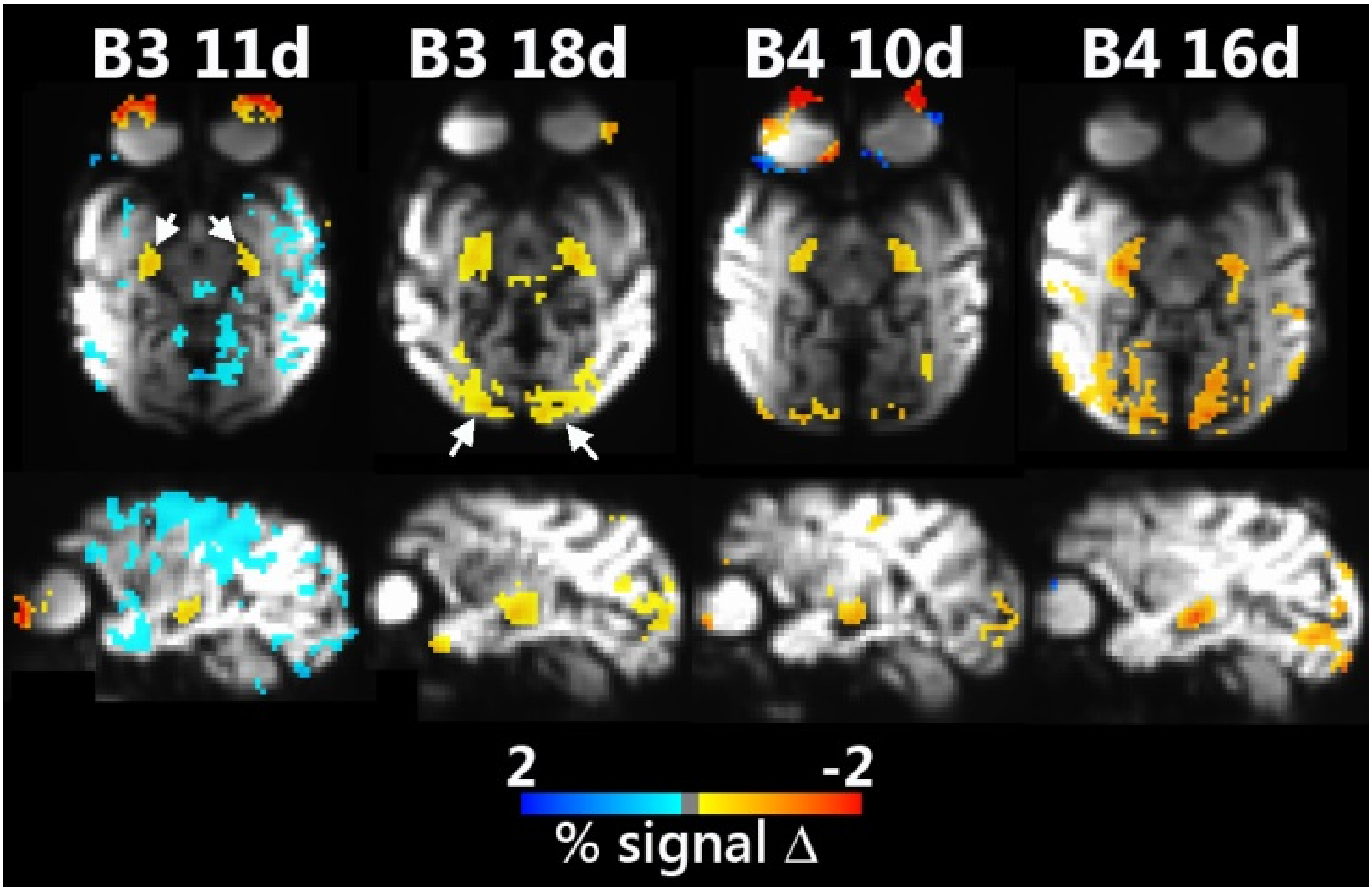

Supplementary Figure 5. Visual Responsiveness before 4 weeks of age. Horizontal (upper row) and parasagittal (lower row) slices showing visual activations in response to blocks of movies minus blocks of gray screen. Maps show beta values thresholded at p<0.05 (FDR corrected, not masked) on the average epi anatomy for each scan session for monkeys B3 and B4 at the ages indicated. In all sessions the LGN (indicated by arrows in the horizontal section for B3 11days) showed strong visual responsiveness, and cortex showed weak activation only in peripheral visual field (arrows in horizontal section for B3 18days).

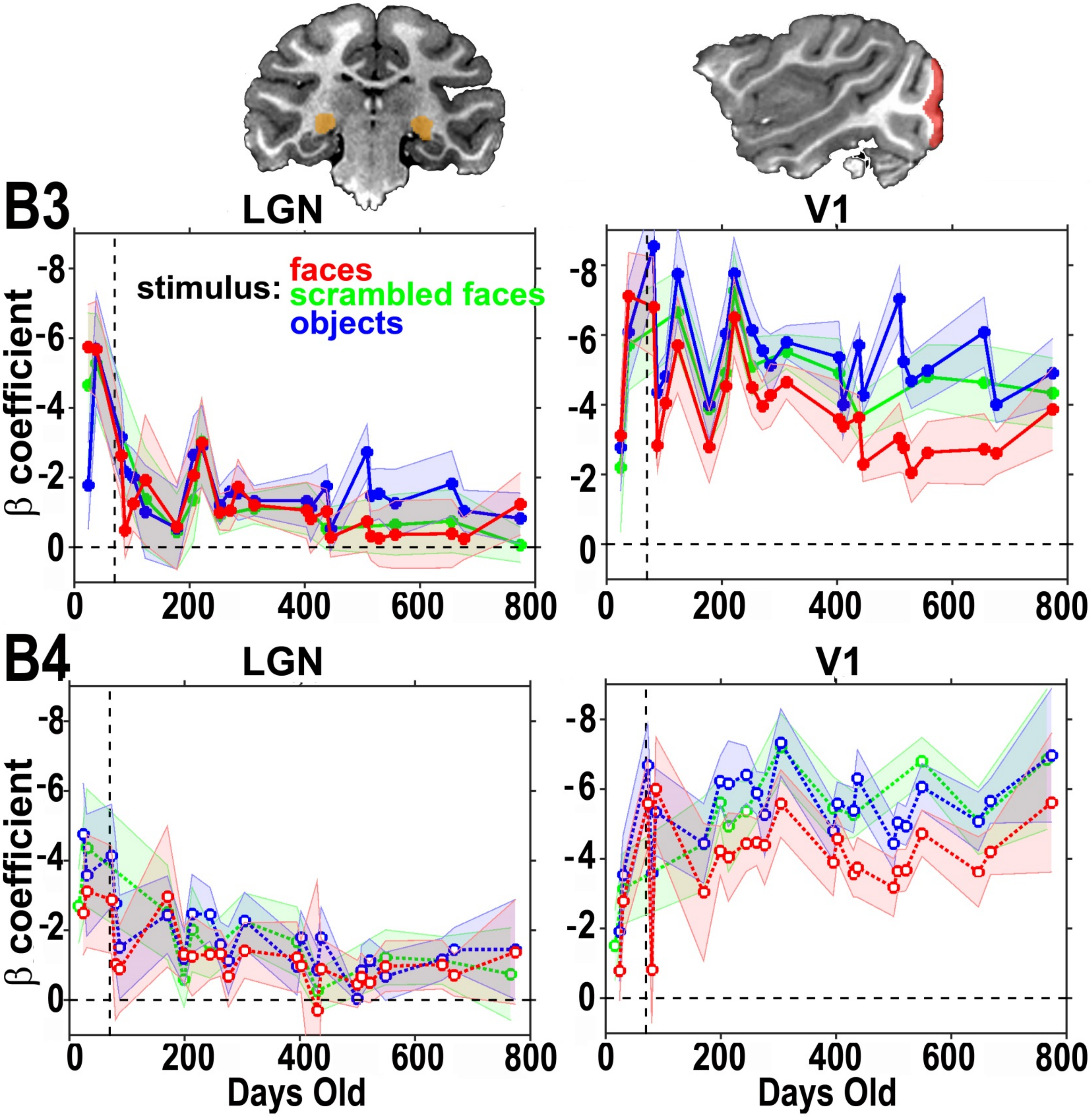

Supplementary Figure 6. Responsiveness to faces (red), objects (blue), and scrambled faces (green) in the LGN and central V1 (central 6-7° of visual field) in monkeys B3 and B4 as a function of age. (For the movie data, red indicates movies including faces, green indicates the same movies with pixelated faces, and blue indicates scene movies.) Shading indicates ±sem. Dotted vertical lines separate movie data from subsequent static image data. Anatomical sections above graphs show the LGN ROI (left, tan) in a coronal section, and the V1 ROI (right, red) in a parasagittal section.

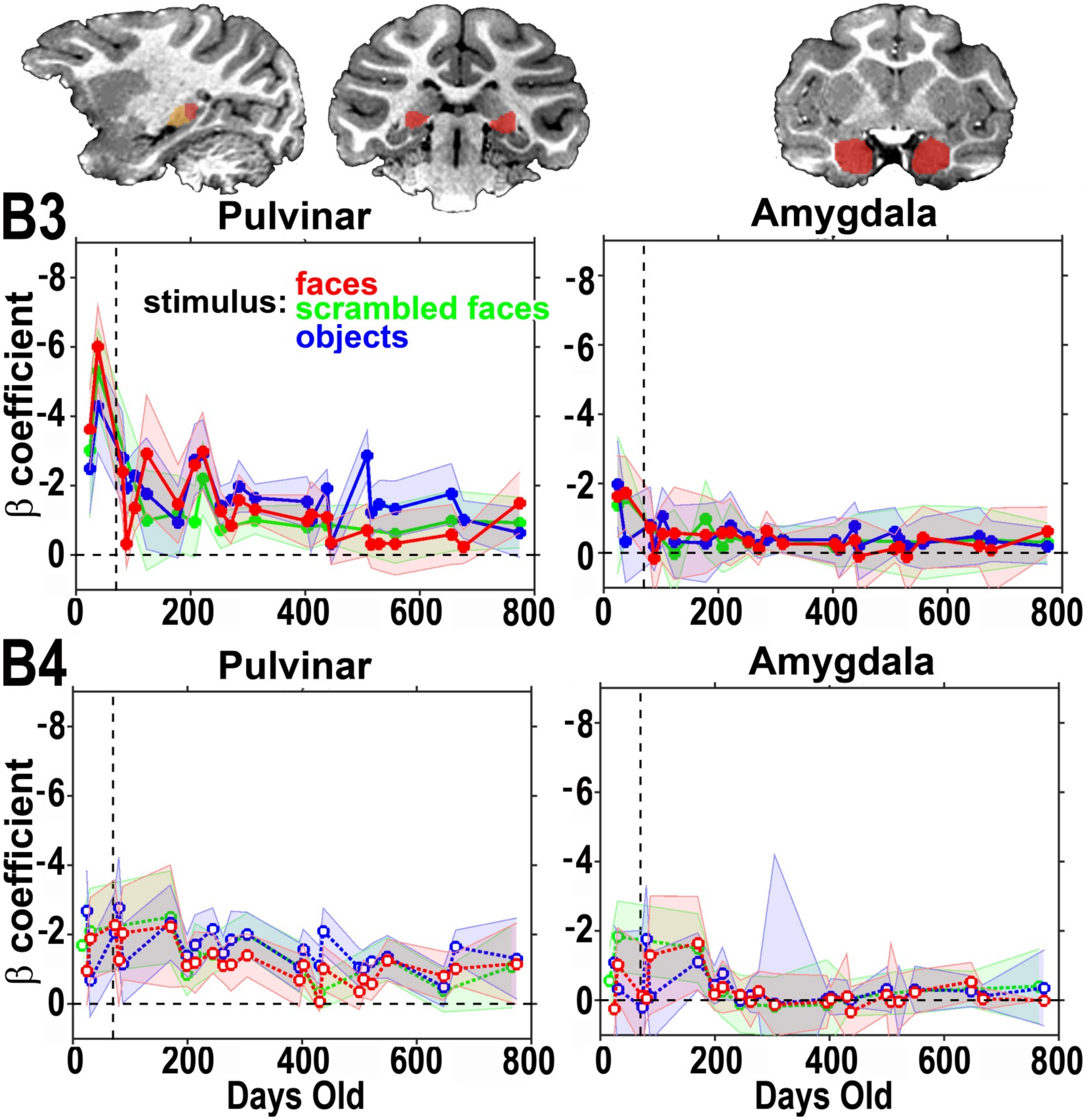

Supplementary Figure 7. Responsiveness to faces (red), objects (blue), and scrambled faces (green) in the ventral pulvinar and amygdala in monkeys B3 and B4 as a function of age. (For the movie data, red indicates movies including faces, green indicates the same movies with pixelated faces, and blue indicates scene movies.) Shading indicates ±sem. Dotted vertical lines separate movie data from subsequent static image data. Anatomical sections above graphs show (L to R) the ventral pulvinar ROI (red) and the LGN ROI (tan) in a parasagittal section, the ventral pulvinar ROI (red) in a coronal section, and the amygdala ROI (red) in a coronal section.

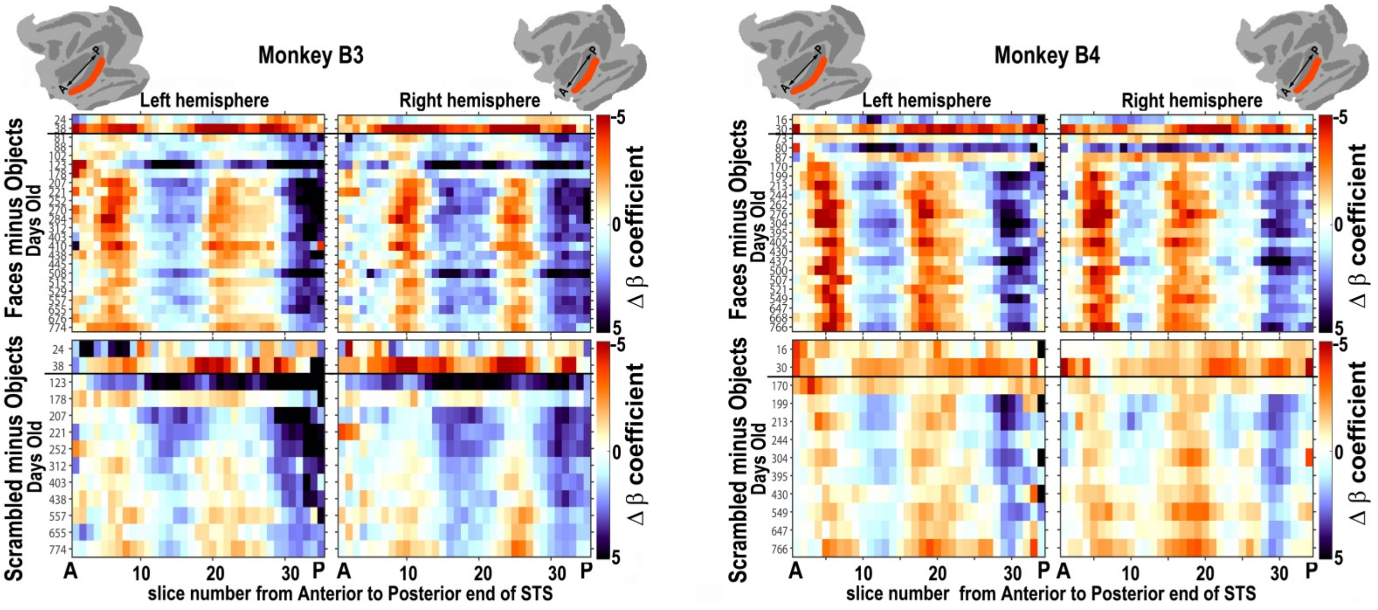

Supplementary Figure 8. Differential responses to faces-minus-objects compared to scrambled-minus-objects along the STS for monkeys B3 and B4. Differences in signal change (beta coefficients) to faces-minus-objects (top matrices) and in signal change to scrambled-minus-objects (bottom matrices) for each 1mm slice of the lower lip of the STS from anterior to posterior (from left to right in each row) for each scan session (vertical dimension) for monkeys B3 and B4. Black line separates early movie data (monkey videos-minus-scene videos) from static image data (faces-minus-objects).

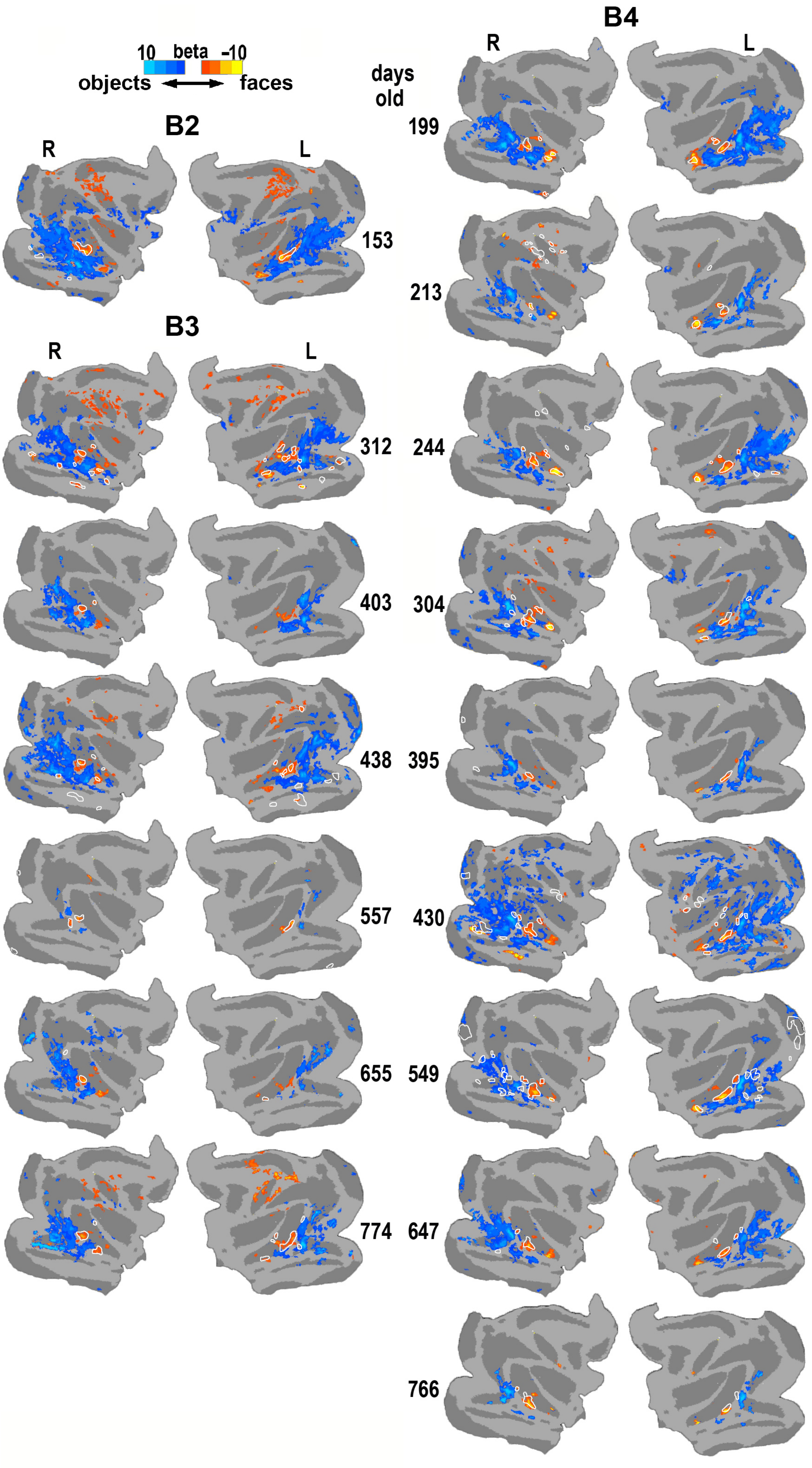
10 beta -10
objects faces
B2
R
L
153
B3
R
L
312
403
438
557
655
774
B4
R
L
days old
199
213
244
304
395
430
549
647
766

Supplementary Figure 9. Comparison of contrast maps for faces-minus-objects to scrambled-minus-objects. White outlines show the location of scrambled >objects thresholded at p<0.05 (FDR corrected) overlaid on the beta coefficient maps from the same scan session of faces-minus-objects, also thresholded at p<0.05 (FDR corrected) for each scan session in which we presented faces, scrambled faces, and objects, and there were significant scrambled>objects activations.

Supplementary References:

1. Arcaro, M.J., Pinsk, M.A., Li, X. & Kastner, S. Visuotopic organization of macaque posterior parietal cortex: a functional magnetic resonance imaging study. *J Neurosci* **31**, 2064-2078 (2011)3074253.
2. Janssens, T., Zhu, Q., Popivanov, I.D. & Vanduffel, W. Probabilistic and single-subject retinotopic maps reveal the topographic organization of face patches in the macaque cortex. *J Neurosci* **34**, 10156-10167 (2014)
3. Kolster, H., Janssens, T., Orban, G.A. & Vanduffel, W. The retinotopic organization of macaque occipitotemporal cortex anterior to V4 and caudoventral to the middle temporal (MT) cluster. *J Neurosci* **34**, 10168-10191 (2014)4115132.
4. Van Essen, D.C. Windows on the brain: the emerging role of atlases and databases in neuroscience. *Curr Opin Neurobiol* **12**, 574-579 (2002)
5. Van Essen, D.C., Drury, H.A., Dickson, J., Harwell, J., Hanlon, D. & Anderson, C.H. An integrated software suite for surface-based analyses of cerebral cortex. *J Am Med Inform Assoc* **8**, 443-459 (2001)131042.
